# *TLR2* 2258 G>A single nucleotide polymorphism and the risk of congenital infection with human cytomegalovirus

**DOI:** 10.1186/s12985-016-0679-z

**Published:** 2017-01-24

**Authors:** Wioletta Wujcicka, Edyta Paradowska, Mirosława Studzińska, Jan Wilczyński, Dorota Nowakowska

**Affiliations:** 1 Scientific Laboratory of the Center of Medical Laboratory Diagnostics and Screening, Polish Mother’s Memorial Hospital - Research Institute, 281/289 Rzgowska Street, Lodz, 93-338 Poland; 2 Department of Perinatology and Gynecology, Polish Mother’s Memorial Hospital - Research Institute, Lodz, Poland; 3 2nd Chair of Obstetrics and Gynecology, Duchess Anna Mazowiecka Public Teaching Hospital, Warsaw, Poland; 4 Laboratory of Molecular Virology and Biological Chemistry, Institute of Medical Biology, Polish Academy of Sciences, Lodz, Poland

**Keywords:** Human cytomegalovirus (HCMV), Toll-like receptor 2 (TLR2), Congenital cytomegaly, Pregnancy, Single nucleotide polymorphism (SNP)

## Abstract

**Background:**

Human cytomegalovirus (HCMV) is responsible for the most common intrauterine infections, which may be acquired congenitally from infected pregnant woman to fetus. The research was aimed to estimate the role of three single nucleotide polymorphisms (SNPs) located in *TLR2* gene, and the common contribution of *TLR2*, and previously studied *TLR4* and *TLR9* SNPs, to the occurrence of congenital HCMV infection in fetuses and newborns.

**Methods:**

The study was performed in 20 Polish fetuses and newborns, congenitally infected with HCMV, and in 31 uninfected controls, as well as with participation of pregnant women, the mothers of 16 infected and 14 uninfected offsprings. Genotypes in *TLR2* SNPs were determined, using self-designed nested PCR-RFLP assays, and confirmed by sequencing. The genotypes were tested for Hardy-Weinberg (H-W) equilibrium, and for their relationship with the development of congenital cytomegaly, using a logistic regression model. The common influence of *TLR2*, *TLR4* and *TLR9* SNPs on the occurrence of congenital disease was estimated by multiple-SNP analysis.

**Results:**

Distribution of the genotypes and alleles in *TLR2* 1350 T>C and 2029 C>T SNPs was similar between the studied groups of fetuses and neonates. In case of 2258 G>A polymorphism, the GA heterozygotic status was significantly more frequent in the infected cases than among the uninfected individuals (25.0% vs. 3.2%, respectively), and increased the risk of HCMV infection (OR 10.00, 95% CI 1.07–93.44; *P* ≤ 0.050). Similarly, the A allele within 2258 G>A polymorphism was significantly more frequent among the infected offsprings than in the uninfected ones (12.5% vs. 1.6%; *P* ≤ 0.050). Complex AA variants for both *TLR2* 2258 and *TLR9* 2848 G>A polymorphisms, were estimated to be at increased risk of congenital HCMV infection (OR 11.58, 95% CI 1.19–112.59; *P* ≤ 0.050). Additionally, significant relationships were observed between the occurrence of complex AA or GA variants for both *TLR2* and *TLR9* SNPs and the increased viral loads, determined in fetal amniotic fluids and in maternal blood or urine specimens (*P* ≤ 0.050).

**Conclusions:**

Among various *TLR2*, *TLR4* and *TLR9* polymorphisms, *TLR2* 2258 G>A SNP seems to be an important factor associated with increased risk of congenital HCMV infection in Polish fetuses and neonates.

## Background

Human cytomegalovirus (HCMV) is responsible for the most common intrauterine infections, transmitted with urine, blood, saliva, genitourinary tract secretions, feces, tears, transplanted organs, and by breastfeeding [1–5]. HCMV infections may be acquired congenitally through vertical transmission of the virus by hematogenous spread from infected pregnant woman to fetus via the placenta or at the time of delivery, when the baby passes through the birth canal [1].

In pregnant women, the seroprevalence rates of the infections were reported to range from 40 to 100% [6–9]. Viral transmissions to the fetus are observed in approximately 30–40% of primarily infected pregnant women, as well as in 1.1–1.7% of the patients with recurrent infections [6–10]. During the first trimester of pregnancy, HCMV infections often result in severe congenital disease, although symptomatic cytomegaly was also observed in the fetuses from pregnant women who acquired HCMV infection during the third trimester of pregnancy [11]. Severe symptoms, diagnosed in about 10 to 15% of congenitally infected neonates, include microcephaly, ventriculomegaly, increased periventricular echogenicity and calcifications [6, 9, 12]. Other symptoms, such as hearing impairment, visual impairment or blindness, difficulties in learning and dyspraxia, observed during the first months or in the first few years of life, were determined in symptomatic congenital cytomegaly, however, they may also occur in a condition, classified as asymptomatic at birth [1, 6, 9].

Several studies reported some involvement of Toll-like receptors (TLRs) in immune response against HCMV [13–15]. Particularly, the TLR2 molecule was shown to support the immunity to the virus [16–18]. In turn, a study with HCMV permissive fibroblasts showed a functional sensing of the virus by TLR2 through direct interaction with viral envelope glycoproteins (gp, g), gB and gH [19]. In human acute monocytic leukemia cell line THP1 and in foreskin fibroblast cell lines, HCMV infection induces the expression of *TLR2*, *TLR3* and *TLR9* genes [15, 18]. In colorectal cancer tissues, HCMV IE1-72 protein expression correlates with TLR2 and TLR4 [20]. The upregulation of *TLR2*/*4* mRNA expression, beside increased levels of IL6 and TNF-α, as well as reduced IL10 expression, were estimated in neutrophils of venous blood samples obtained from pregnant women with early-onset preeclampsia with hemolysis, elevated liver enzymes and low platelets syndrome that had significantly higher anti-HCMV IgG seropositivity, as compared to non-pregnancy controls [21]. In platelets, extracted from healthy donors, a purified clinical HCMV isolate VR1814 bound the TLR2 molecule, and activated signal transduction, degranulation and release of CD40 IL1β and VEGF [22]. The involvement of TLR2, as well as of IFN-β, in the response to HCMV, was also reported for NK cells [17].

Taking into account genetic modifications, the single nucleotide polymorphisms (SNPs) located within *TLR2* gene, were reported to be correlated with HCMV infections as well [23–25]. In children with congenital HCMV disease, the CC genotype in *TLR2* 1350 T>C SNP (rs3804100) was associated with the infection, although no relationship was established with the course of cytomegaly [25]. In liver transplant recipients, the homozygotic status in *TLR2* 2258 G>A SNP (rs5743708) was associated with HCMV disease, especially tissue-invasive disease [24]. In another study, also performed in liver transplant recipients, significantly higher viral loads were observed among the patients, either with the minor alleles or being heterozygotes in *TLR2* 2258 G>A polymorphic site, as compared to wild type homozygotes [26]. An in vitro study with transfected HEK293 cells showed some involvement of *TLR2* 2258 G>A SNP in the TLR2 signaling pathway after exposure to viral gB protein [23]. Therefore, the analyzed polymorphism was suggested to participate in the development of HCMV disease in humans [23]. Other studies also showed the SNPs located in *TLR2*, as well as in *TLR3*, *TLR4*, *TLR7* and *TLR9* genes, to be contributing to HCMV infection [13, 14, 27]. So far, no study has shown any relationship between genetic alterations in *TLR2* 2258 G>A SNP and congenital HCMV infection. Several studies had previously been performed to investigate the role of this polymorphism in pregnancy disorders, including preeclampsia, bacterial vaginosis, and preterm birth [28–31]. Moreover, the GA heterozygosity within *TLR2* 2258 polymorphism was shown to be involved in tuberculosis among Turkish children, as well as *Candida* sepsis in German adult patients [32, 33].

We previously reported a possible contribution of *TLR4* and *TLR9* SNPs to congenital cytomegaly [14]. These recently published outcomes, as well as the available literature data on the contribution of *TLR2* SNPs to the occurrence of HCMV infection, prompted us to undertake further research, evaluating the role of *TLR2* 1350 T>C coding synonymous (Ser450, rs3804100), as well as 2029 C>T (Arg677Trp, rs121917864) and 2258 G>A non-synonymous (Arg753Gln) SNPs in the development of HCMV congenital infection in fetuses and neonates. Moreover, the common influence of *TLR2* 2258 G>A, as well as of the recently studied *TLR4* 896 A>G, 1196 C>T and *TLR9* 2848 G>A SNPs [14] on the occurrence of the infection and on congenital cytomegaly development was also estimated. The distribution of genotypes and alleles in *TLR4* and *TLR9* polymorphisms, and of the haplotypes for *TLR4* SNPs, between the analyzed groups of the offsprings, was reported in our previous paper [14].

## Methods

The reported study included 20 fetuses and neonates on the day of birth, congenitally infected with HCMV, and 31 control cases without infection. In the analyzed population of infected offsprings, 35.0% (7/20) were fetuses and 65.0% (13/20) - neonates. Among the studied patients, 18 HCMV infected fetuses and neonates, as well as 20 control individuals, were previously investigated regarding *TLR4* and *TLR9* SNPs [14]. Considering pregnant women, the mothers of 16 infected fetuses and neonates, and of 14 uninfected offsprings, were also enrolled into the reported study, based on the availability of clinical samples. Fetal, neonatal, and maternal specimens were retrospectively, randomly collected at the Department of Fetal-Maternal Medicine and Gynecology of the Polish Mother’s Memorial Hospital - Research Institute (PMMHRI) in Lodz between the years 2000 and 2013. Among the infected offsprings, eleven (11) presented symptomatic cytomegaly, while nine (9) were asymptomatic. The ultrasound markers, associated with symptomatic disease consisted of ventriculomegaly, hydrocephaly and fetal hydrops, as well as demonstrated intrauterine growth restriction (IUGR), ascites, pericardial effusion, cardiomegaly and hyperechogenic foci in various organs. In turn, asymptomatic cytomegaly was determined in fetuses and newborns without any ultrasound symptoms, which could have been related to the disease. The materials, classified for genetic studies, included amniotic and/or ascitic (two samples) fluids, umbilical cord blood and amniotic membranes, as well as whole blood, plasma, serum and urine samples from newborns. The fetal amniotic fluid samples were obtained via amniocentesis in pregnant women, treated at the Institute. The umbilical cord blood samples and membranes of fetuses, as well as neonate blood and urine samples were collected on the day of birth. A preliminary diagnosis of intrauterine HCMV infection was based on the maternal serological status and fetal and neonatal cytomegaly-related ultrasound markers. HCMV DNA finding in, at least, one of the available examined clinical materials obtained from a single patient, was the confirmation of congenital infection. Both detection and quantitation of HCMV DNA, was also performed for 75.0% (15/20) of mothers of the infected offsprings. In case of pregnant women, whole blood, serum or urine samples were used to determine the presence and the levels of viral DNA. The study was approved by the Research Ethics Committee at the PMMHRI. The clinical samples, used in the study, were previously collected and anonymized. Informed consent forms were signed by the pregnant women, participating in the study, and the consent procedure was accepted by the Research Ethics Committee.

### Serological tests

Blood specimens were collected from randomly selected pregnant women by venipuncture during their first visit to the Institute. Serum samples were obtained by centrifugation and then stored at 4 °C before analysis, on the day of blood collection. Serological tests were performed at the Department of Clinical Microbiology at the Institute.

Screening for anti-HCMV antibodies was performed with Eti-Cytok G-Plus and Eti-Cytok M-Reverse Plus tests (Diasorin/Biomedica, Italy), between the years 2000 and 2001, VIDAS CMV IgG and IgM tests (bioMérieux, France) – between 2001 and 2006, anti-CMV IgG and IgM tests (Diasorin/Biomedica, Italy) – between 2006 and 2011 years, and ELFA assays – from the year 2012. HCMV infection was determined in pregnant women in case of IgG seroconversion during pregnancy in the presence of IgG and IgM specific antibodies or a low IgG avidity index. Active viral infection was determined in the pregnant women, as well as in their fetuses and neonates, using real-time Q PCR assays for viral *UL55* gene in blood, urine and amniotic fluids.

### DNA isolation

Genomic and/or viral DNA was extracted from 5 ml of the amniotic fluid, 3 ml of the ascitic fluid, 200 μl of umbilical cord blood, neonatal whole blood, plasma or serum specimens, from fetal membranes and 5 ml of neonatal urine specimens, as well as from maternal whole blood, serum or urine samples, using a QIAamp DNA Mini Kit (QIAGEN, Hilden, Germany). The extracted DNA was diluted in 100 μl of elution buffer and stored at −20 °C until molecular analyses.

### Detection and quantification of HCMV DNA

HCMV DNA was identified and quantified by a real-time Q PCR assay of a viral *UL55* gene fragment of 150 bps in length, as previously described [34, 35]. Standard curves, used in the quantitative analyses, were plotted with serial 10-fold dilutions from 10^5^ to 1 plasmid DNA, containing the entire HCMV *UL55* open reading frame [36]. The real-time Q PCR assays were performed, using a 7900 HT Fast Real-Time PCR System (Applied Biosystems, USA).

### Determination of SNPs located within *TLR2* gene

**Fig. 1:**
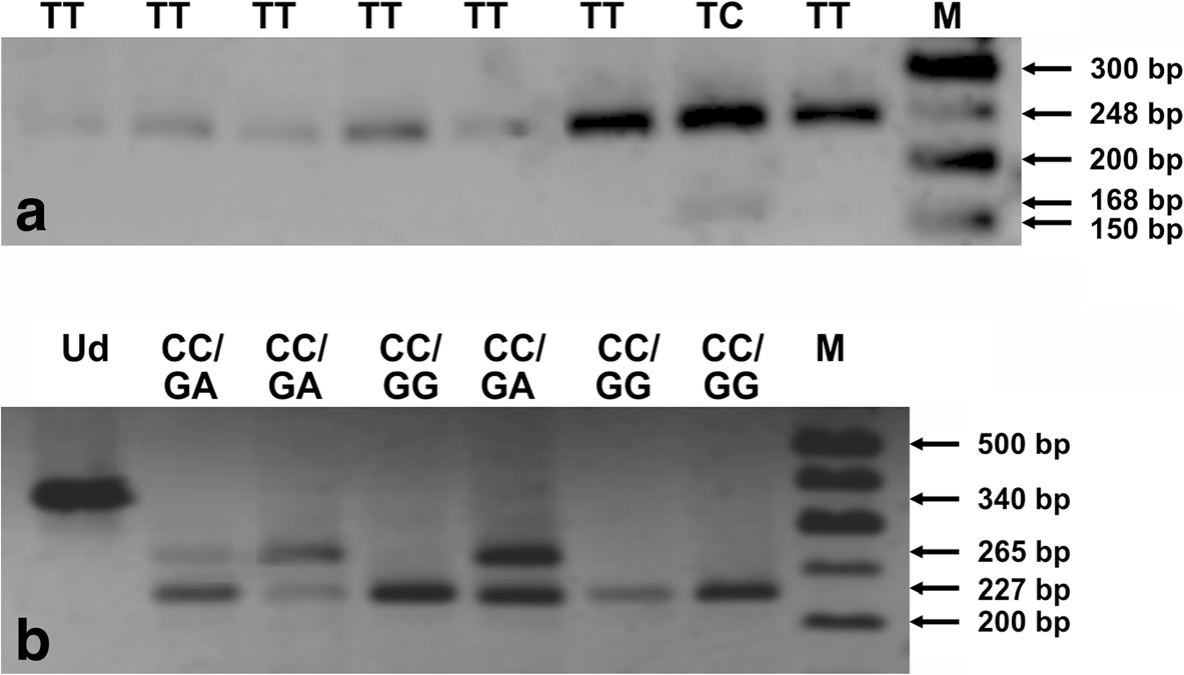
PCR-RFLP profiles for *TLR2* 1350 T>C (**a**), and 2029 C>T and 2258 G>A (**b**) SNPs. RFLP products were separated in 2% agarose gels, stained with ethidium bromide. The numbers on the right side of electropherograms show the lengths of resolved DNA fragments. M – 50 bp DNA marker; Ud – undigested PCR product; CC, GG, GA, TC, and TT – genotypes in analyzed *TLR2* SNPs

**Fig. 2:**
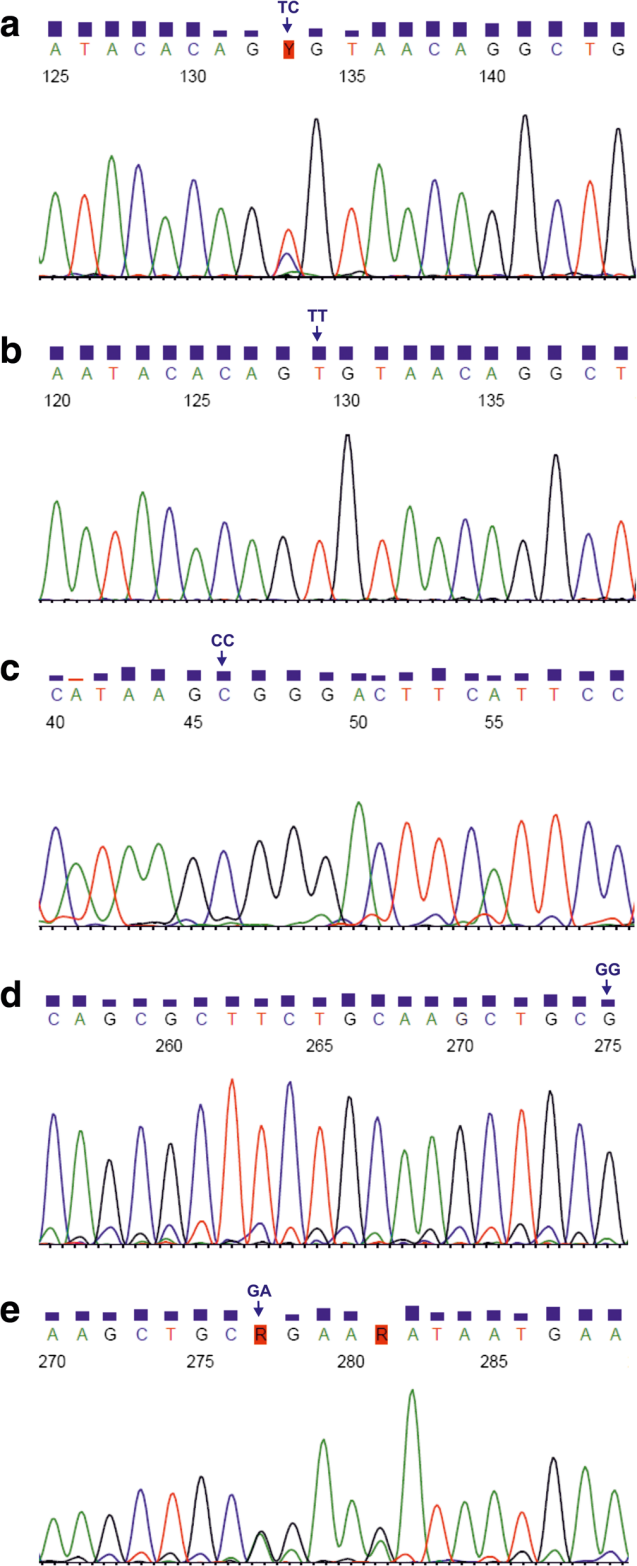
Chromatograms comprising *TLR2* 1350 T>C (**a**, **b**), 2029 C>T (**c**), and 2258 G>A (**d**, **e**). The genotypes in *TLR2* SNPs were determined for the forward strand sequences. CC, GG, GA, TC, and TT – genotypes in described SNPs

Nested PCR assays were developed to determine the genotypic status in *TLR2* 1350 T>C, 2029 C>T, and 2258 G>A polymorphic sites. The GenBank accession numbers for the coding sequences, as well as the sequences of external and internal primers, amplicon lengths and annealing temperatures, used in nested PCRs, are shown in Table 1. The external primers were designed, using the Vector NTI Suite 5.5 software, whereas the internal primers were taken from published articles [37–40]. Nested PCR assays were performed, using a HotStarTaq® Master Mix Kit (QIAGEN, Hilden, Germany). The amplification conditions were as follows: an initial, 15-min activation at 95 °C and 40 cycles of repeated denaturation at 94 °C for 30 s, annealing at 52 °C (for external primers) or 55 °C/59 °C (for internal primers encompassing 1350 T>C/ 2029 C>T and 2258 G>A SNPs, respectively) for 1 min and extension at 72 °C for 2 min and the final extension at 72 °C for 10 min. The nested PCR products for *TLR2* 1350 T>C, or 2029 C>T and 2258 G>A SNPs, were resolved by electrophoresis on 1% agarose gels and then digested with MwoI or AciI endonucleases, respectively. Restriction reaction mixtures consisted of 10 μl of the PCR product, 10 U of endonuclease, 1 x concentrated buffer for the enzyme and distilled nuclease-free water, added to the final reaction volume of 20 μl. The digestions were performed overnight at 37 °C, and then the MwoI or AciI enzymes were inactivated by incubation for 20 min at 80 °C or 65 °C, respectively, following the restriction reaction. The obtained restriction products were resolved on 2% agarose gels. The genotypes in *TLR2* polymorphic sites were determined for all the analyzed samples, based on the length of restriction fragments ([37–39], see Table 2, Fig. 1). The genotypes were studied at the Scientific Laboratory of the Center of Medical Laboratory Diagnostics and Screening. The randomly selected PCR products, representative for six TT homozygotes and one TC heterozygote in *TLR2* 1350 T>C polymorphism, for 39 CC homozygotes in 2029 C>T locus, as well as for 33 GG homozygotes and six GA heterozygotes in 2258 G>A SNP, were confirmed by sequencing by the Sanger method at the Genomed Joint-Stock Company (Warsaw, Poland). The exemplary chromatograms with fragments of DNA sequences for both kinds of determined genotypes in the analyzed polymorphic sites are presented in Fig. 2. The sequenced and the reference fragments of *TLR2* gene were compared, using the BLASTN program and the chromatograms were analyzed, using the Sequence Scanner 1.0 (Applied Biosystems) software.

**Table 1:** Primers, annealing temperatures and amplicons used in PCR assays for SNPs in the *TLR2* gene

Gene	GenBank accession no.^a^	SNP^b^ name	Primer sequences (5′-3′)		Annealing temperature [°C]	Amplicon length (bps)^c^
*TLR2*	NC_000004.12	1350 T>C	External	For: AATTCAGCCTGTGAGGATGC	52	361
		(rs3804100)		Rev: GTAAGAGGGAGGCATCTGGTA		
		(Ser450)	Internal	For: TCATTTGGCATCATTGGAAA	55	248
		(S450)		Rev: GAGTTGCGGCAAATTCAAAG		
		2029 C>T; 2258 G>A	External	For: CGGAATGTCACAGGACAGC	52	605
		(rs121917864; rs5743708)		Rev: GGACTTTATCGCAGCTCTCAG		
		(Arg677Trp; Arg753Gln)	Internal	For: GCCTACTGGGTGGAGAACCT	59	340
		(R677W; R753Q)		Rev: GGCCACTCCAGGTAGGTCTT		

^a^ *No.* number

^b^ *SNP* single nucleotide polymorphism

^c^ *bps* base pairs

**Table 2:** Lengths of restriction fragments and genotypic profiles

*TLR2* SNP^a^	Restriction enzyme	Profile (bps)^b^
1350T>C	MwoI	TT: 248
		TC: 248, 164, 84
		CC: 164, 84
2029 C>T	AciI	CC: 227, 75, 38
		CT: 302, 227, 75, 38
		TT: 302, 38
2258 G>A	AciI	GG: 227, 75, 38
		GA: 227, 265, 75, 38
		AA: 265, 75

^a^ *SNP* single nucleotide polymorphism

^b^ *bps* base pairs

### Statistical analysis

The prevalence rates of genotypes and alleles in *TLR2* 2258 G>A SNP were calculated, both in the HCMV infected and uninfected fetuses and neonates by means of descriptive statistics. The offsprings were studied for the Hardy-Weinberg (H-W) equilibrium, using the SNPStats software (http://bioinfo.iconcologia.net/en/SNPStats_web). The applied test compares the observed and expected allele prevalence rates under the assumption of independence, as well as estimates a Chi-squared distribution with one degree of freedom. A relationship was determined between the genetic modifications in *TLR2* polymorphism and the development of congenital HCMV infection, using cross-tabulation, Pearson’s Chi-squared and Fisher’s exact tests, as well as the logistic regression model. An association was estimated, based on multiple-SNP analysis by the Expectation Maximization (EM) algorithm, between the genetic status within *TLR2* 2258 G>A, as well as in previously studied *TLR4* 896 A>G, 1196 C>T and *TLR9* 2848 G>A SNPs [14] and the occurrence of HCMV infection and development of congenital cytomegaly. The linear regression model was used to calculate a correlation of the fetal and neonatal genotypic status in *TLR2* 2258 G>A polymorphism with HCMV DNA levels in body fluids of the studied offsprings or their mothers. All the results were defined as statistically significant, when attained the significance level of *P* ≤ 0.050. A part of the statistical analysis of the distribution of identified alleles between studied groups was supported by the NCSS 2004 software.

## Results

### Hardy-Weinberg equilibrium

In the fetuses and newborns, which were either congenitally HCMV infected or uninfected, the genotypes in *TLR2* 2258 G>A polymorphism preserved the H-W equilibrium (*P* = 1.000).

### HCMV DNA loads in fetal, neonatal and maternal body fluids

The median load of HCMV DNA in whole blood specimens of the infected offsprings was 5.9 × 10^3^ copies/ml and ranged from 1.6 × 10^2^ to 2.0 × 10^6^ copies/ml, while the mean viral load was 2.8 × 10^5^ copies/ml. Regarding amniotic fluid samples, the median HCMV DNA load was 1.5 × 10^3^ copies/ml, ranging from 2.2 × 10^2^ to 4.3 × 10^6^ copies/ml, and the mean viral load was 6.7 × 10^5^ copies/ml. In case of ascitic fluids, the median and mean viral loads were 3.4 × 10^3^ copies/ml, and ranged from 1.5 × 10^2^ to 6.7 × 10^3^ copies/ml. HCMV DNA load determined for plasma specimen was 2.1 × 10^2^ copies/ml, and for urine samples – 9.3 × 10^5^ copies/ml. In pregnant women, the median viral load in whole blood specimens was 6.3 × 10^2^ copies/ml and the mean viral load was 4.1 × 10^3^ copies/ml. In urine samples, median HCMV load was 4.3 × 10^2^ copies/ml and mean load was 9.3 × 10^2^ copies/ml. In case of plasma samples, the median and mean HCMV loads were 3.5 × 10^2^ copies/ml, while the serum viral load was 3.6 × 10^3^ copies/ml.

### Prevalence rates of the genotypes in *TLR2* 1350 T>C, 2029 C>T and 2258 G>A SNPs

Genotypes in *TLR2* 1350 T>C, 2029 C>T, and 2258 G>A SNPs, were successfully determined for all DNA samples obtained from fetuses and newborns selected for genetic tests. In case of *TLR2* 1350 T>C SNP, only one uninfected fetus carried TC heterozygotic status, while all other fetuses and newborns were TT homozygotes. In the range of *TLR2* 2029 C>T polymorphism, all the studied offsprings were CC homozygotes. Considering *TLR2* 2258 G>A SNP, the prevalence rates of GG and GA genotypes were 75.0% (15/20) and 25.0% (5/20), respectively (see Table 3A). In case of the uninfected control offsprings, the prevalence rates of GG and GA genotypes in *TLR2* SNP were 96.8% (30/31) and 3.2% (1/31), respectively. The GA heterozygotic status in the SNP, was significantly more frequent among the infected than the uninfected cases (25.0% vs. 3.2%, respectively), and increased the risk of HCMV infection (OR 10.00, 95% CI 1.07–93.44; *P* ≤ 0.050). Among the patients with symptomatic cytomegaly, the prevalence rates of GG and GA genotypes were 81.8% (9/11) and 18.2% (2/11), respectively (see Table 3B). In case of asymptomatic disease, the GG and GA genotype carriers were identified in 66.7% (6/9) and 33.3% (3/9) of cases, respectively. Regarding the congenital cytomegaly outcome, similar prevalence rates of the genotypes were observed in the infected offsprings with symptomatic and asymptomatic disease. Among the pregnant women, being mothers of both HCMV infected and uninfected fetuses and neonates, only one mother of the infected symptomatic offspring, carried GA heterozygotic status in *TLR2* 2258 G>A locus, while all other studied women demonstrated GG homozygotes within this polymorphism. Taking into account the outcomes for *TLR2* 2258 G>A SNP, obtained in the current study, as well as for *TLR4* 896 A>G, 1196 C>T and *TLR9* 2848 G>A SNPs, reported in our previous paper [14], the additional multiple-SNP analysis showed the occurrence of complex AA variants in the range of both *TLR2* and *TLR9* polymorphisms to be correlated with an increased risk of HCMV infection among studied fetuses and neonates (OR 11.58, 95% CI 1.19–112.59; *P* ≤ 0.050, see Table 4). In case of other multiple-SNP variants, a similar distribution pattern was observed between the infected and uninfected fetuses and neonates. Moreover, the distribution of all the multiple-SNP variants, determined between the infected offsprings with both symptomatic and asymptomatic cytomegaly was also similar.

**Table 3:** Single-SNP analysis of the relationship between *TLR2* 2258 G>A polymorphism and congenital HCMV infection

A.				
Genotype	Genotype frequencies; *n* (%)^a^		OR^b^ (95% CI)^c^	*P*-value^d^
	Infected cases	Controls		
GG	15 (75.0%)	30 (96.8%)	1.00	0.018
GA	5 (25.0%)	1 (3.2%)	10.00 (1.07–93.44)	
B.				
Genotype	Genotype frequencies; *n* (%)^a^		OR^b^ (95% CI)^c^	*P*-value^d^
	Symptomatic cases	Asymptomatic cases		
GG	9 (81.8%)	6 (66.7%)	1.00	0.440
GA	2 (18.2%)	3 (33.3%)	0.44 (0.06–3.51)	

The prevalence rates of genotypes in *TLR2* SNP were compared between infected and uninfected fetuses and newborns (A) as well as symptomatic and asymptomatic offsprings with congenital cytomegaly (B)

*P* ≤ 0.050 is considered as significant

^a^ *n* number of tested fetuses and newborns

^b^ *OR* odds ratio

^c^ *95% CI*, confidence interval

^d^logistic regression model

**Table 4:** Multiple-SNP variants for *TLR2*, *TLR4* and *TLR9* polymorphisms and the occurrence of congenital HCMV infection

*TLR* gene’s polymorphisms	Multiple-SNP^a^ variant	Prevalence rates of multiple-SNP variants		OR^b^ (95% CI^c^)	*P*-value^d^
		Infected cases	Uninfected controls		
*TLR2* 2258 G>A – *TLR4* 896 C>T	GA	0.850	0.874	1.00	
	GG	0.025	0.109	0.39 (0.06–2.59)	0.330
	AA	0.125	0.016	8.81 (0.93–83.12)	0.063
*TLR2* 2258 G>A – *TLR4* 1196 C>T	GC	0.875	0.859	1.00	
	AC	0.125	0.016	8.03 (0.85–75.63)	0.075
*TLR2* 2258 G>A – *TLR9* 2848 G>A	GA	0.400	0.576	1.00	
	GG	0.475	0.408	1.62 (0.58–4.53)	0.360
	AA	0.125	0.016	11.58 (1.19–112.59)	0.040

*P* ≤ 0.050 is considered as significant

^a^ *SNPs* single nucleotide polymorphisms

^b^ *OR* odds ratio

^c^ *95% CI* confidence interval

^d^logistic regression model

### Prevalence rates of the alleles in *TLR2* 2258 G>A polymorphism

In HCMV infected fetuses and neonates, the prevalence rates of G and A alleles in *TLR2* 2258 G>A SNP, were 87.5% (35/40) and 12.5% (5/40), respectively (see Table 5A). Among the uninfected control cases, the corresponding values of G and A alleles were 98.4% (61/62) and 1.6% (1/62), respectively. The A allele was significantly more frequently observed among the infected offsprings, when compared to the uninfected ones (12.5% vs. 1.6%; *P* ≤ 0.050; Fisher’s exact test). Among the symptomatic patients, the prevalence rates of G and A alleles were 90.9% (20/22) and 9.1% (2/22), respectively (see Table 5B). In case of the asymptomatic disease, the corresponding values of G and A alleles were 83.3% (15/18) and 16.7% (3/18), respectively. Similar prevalence rates of the alleles were observed in the symptomatic and the asymptomatic cases (*P* = 0.471; Pearson’s Chi-squared test).

**Table 5:** Distribution of the alleles, located in *TLR2* 2258 G>A polymorphic site

A.			
Gene polymorphism and allele	No.^a^ of carriers with *TLR2* alleles (%)		*P*-value
	Infected cases	Controls	
*TLR2* 2258 G>A			
G	35 (87.5)	61 (98.4)	0.033^b^
A	5 (12.5)	1 (1.6)	
B.			
Gene polymorphism and allele	No.^a^ of carriers with *TLR2* alleles (%)		*P*-value
	Symptomatic cases	Asymptomatic cases	
*TLR2* 2258 G>A			
G	20 (90.9)	15 (83.3)	0.471^c^
A	2 (9.1)	3 (16.7)	

The prevalence rates of alleles in *TLR2* SNP were compared between infected and uninfected fetuses and newborns (A) as well as between symptomatic and asymptomatic offsprings with congenital cytomegaly (B)

*P* ≤ 0.050 is considered significant

^a^ *No.* number

^b^Fisher’s exact test

^c^Pearson’s Chi-squared test

### Relationship between genotypes in *TLR2* 2258G>A SNP and HCMV DNA load

In the fetuses and neonates, the genotypic status within *TLR2* 2258 G>A SNP was not associated with HCMV DNA loads, determined both in whole blood and amniotic fluid samples (*P* = 0.460 and *P* = 0.250, respectively). Among the infected offsprings, the GA heterozygotes tended to be correlated with higher viral loads in whole blood and urine samples of their mothers (mean difference (MD) 1.1 × 10^4^ copies/ml; *P* = 0.180 and *P* = 0.200, respectively). Multiple-SNP analysis showed GA complex variants in the range of *TLR2* 2258 and *TLR9* 2848 G>A SNPs to be correlated with higher viral loads, determined in fetal amniotic fluids and maternal urine samples (MD 1.2 × 10^6^ and 1.1 × 10^4^ copies/ml, respectively, *P* ≤ 0.050). In case of AA multiple-SNP variants for the studied *TLR2* and *TLR9* SNPs, significantly higher viral loads were estimated in maternal blood and urine specimens (MD 8.5 × 10^4^ and 1.5 × 10^4^ copies/ml, respectively, *P* ≤ 0.050).

## Discussion

In the reported study, the GA heterozygotic status in *TLR2* 2258 G>A SNP was found to be correlated with HCMV congenital infection in Polish fetuses and newborns. The identified heterozygotes in the range of *TLR2* SNP were estimated as 10 times more susceptible to develop the infection. TLR2 molecule was previously reported to be involved in the immune response against HCMV [15, 19–21]. In ectocervical tissue, HCMV infection was blocked by ligands for TLR2 (LTA), as well as TLR9 (CpG) molecules [41]. In HCMV infected human permissive fibroblasts, the TLR2 molecule was reported to have been involved in NF-κB activation and inflammatory cytokine secretion, but not in IFN signaling [16]. In turn, the TLR2 molecule of murine monocytes was shown to be involved in the production of IFN-β, observed after stimulation with murine cytomegalovirus (MCMV) or vaccinia virus [VV; [41]). TLR2 knock-out mice were reported to have impaired NK cell function and elevated MCMV load [42]. The TLR2 molecule was also observed as involved in the immune response to other viruses, such as varicella zoster virus (VZV), Epstein-Barr virus (EBV) and murine respiratory syncytial virus (RSV), as well as hepatitis B and C viruses [43–47].

Taking into account genetic modifications within the *TLR2* gene, previous studies also reported contribution of *TLR2* 2258 G>A coding non-synonymous SNP to HCMV infection, although the mutated homozygotes were found in some studied populations only [23, 24, 26]. In a cohort of liver transplant recipients treated at the Mayo Clinic, Minnesota, United States, some relationship was determined between homozygosity in the analyzed *TLR2* polymorphism and HCMV infection, especially in tissue-invasive disease [24]. An in vitro study of the transfected human embryonic kidney (HEK) 293 cells, that were exposed to HCMV gB, showed *TLR2* 2258 SNP to have been involved in TLR2 signaling impairment [23]. Another study, performed in liver transplant recipients with chronic hepatitis C, treated also at the Mayo Clinic, showed a certain association of *TLR2* polymorphism with HCMV load [26]. The homozygotic status in the analyzed SNP was correlated with cytomegaly, as well as with an increased risk for the disease after adjusting for patienťs age, HCMV serostatus and allograft rejection [26]. Taking into account our results and the previous literature data, *TLR2* 2258 G>A SNP may be involved in congenital infection with HCMV in Polish fetuses and neonates. Considering HCMV DNA loads determined in fetal and neonatal body fluids, no association was observed with genotypic variability within the analyzed polymorhism. Before our study, no attempt had been reported to investigate the function of *TLR2* 2258 G>A polymorphism in congenital infection with HCMV. A study performed in 88 infants and 63 adults infected postnatally with HCMV, and in 28 healthy neonates and 50 healthy adults, may suggest a possible protective role of CT heterozygotic status in *TLR2* 2029 C>T locus against the infection development among adult patients [13]. However, similarly to our outcomes, the mentioned study showed that all the analyzed infants were CC homozygotes in the range of 2029 C>T polymorphism [13]. Additionally, we also found the same distribution of genotypes within *TLR2* 1350 T>C SNP among both HCMV infected and uninfected fetuses and neonates, although the polymorphism was reported to be significantly associated with congenital cytomegaly among Japanese children [25]. Since the C allele in *TLR2* 1350 T>C SNP is more frequent among the Japanese than in the European populations (25.48% vs. 6.36%, see http://www.ncbi.nlm.nih.gov/variation/tools/1000genomes/?q=rs3804100), the geographical origin might be the major reason of differential role of the analyzed polymorphism in susceptibility to congenital infection with HCMV. It seems that further studies with larger groups of patients, congenitally infected with the virus, would be an interesting challenge. Considering *TLR2* 2258 G>A SNP, the individual cases of GA heterozygotes and AA recessive homozygotes were observed among HCMV infected infants, and not among the uninfected offsprings [13]. Similarly, in our study, the prevalence rate of the minor allele in *TLR2* 2258 G>A locus was higher among the HCMV infected infants than in the uninfected ones. Moreover, the paper by Jabłońska et al. reported the occurrence of AA homozygotes among the infected adults, but not among uninfected patients [13]. The GA heterozygotes were not found among non-HCMV infected infants [13]. In our study, we observed minor alleles in *TLR2* 2258 SNP only as heterozygotes, whereas no AA homozygotes were found. It should be emphasized that in our reported study, we evaluated fetuses and neonates on the day of birth, with congenital HCMV infection. In contrast, Jabłońska et al. explored infants, aged 1–12 months, with postnatal HCMV infection, qualified by clinical symptoms or evidence of the infection confirmed by viral DNA detection in whole blood/urine samples after 3 weeks of life, and by the presence of HCMV-specific antibodies [13]. It is possible that the obtained results are different, since the two different groups of patients were examined. Additionally, the prevalence rates of genotypes and alleles, located within the analyzed *TLR2* polymorphisms, as well as their associations with the occurrence of congenital HCMV infection, estimated in the current research, might have been due to the small sample size cohort of the studied offsprings. Likewise to our results, the prevalence rates of A allele in *TLR2* 2258 G>A SNP was determined to be low and a lack of AA homozygotes was also reported in other study groups, such as German, Finnish and Caucasian adults, or Turkish children [32, 48–50]. The heterozygotic status and A allele in *TLR2* 2258 G>A polymorphism were significantly more frequently identified among Turkish children with tuberculosis (TB) than in control cases [32]. In addition, the prevalence rate of *TLR2* 2258 SNP was reported to be increased in patients with pulmonary TB alone, as well as with definitive pulmonary plus extrapulmonary TB, as compared to cases with latent TB infection [32]. In German adult patients with *Candida* sepsis, the heterozygotic status in *TLR2* 2258 G>A SNP was correlated with altered cytokine release, including increased plasma concentrations of TNF-α and decreased levels of IFN-γ and IL8 [33]. Considering genotypic variability in *TLR2* 2258 locus, it should also be noticed that some populations, living in Barbados, the South-Western USA, Bangladesh, China, Nigeria, Texas, Gambia, Japan, the United Kingdom, Vietnam, Kenya, Sierra Leone, Los Angeles in the USA, as well as in Lima, Peru (see http://www.ncbi.nlm.nih.gov/variation/tools/1000genomes/?q=rs5743708) do not possess the minor A allele within the reported region. Hence the polymorphism, analyzed in this study, can plausibly be involved in the development of congenital infection with HCMV only in some populations. Given the previous papers on the role of TLR2 molecule in the immune response against HCMV, the altered *TLR2* gene in the range of 2258 G>A SNP may also be involved in the development of congenital infection with the virus in populations, carrying the minor allele. Additionally, previous papers from studies, performed in fetuses and neonates, as well as in children with congenital HCMV infection, also showed some contribution of other *TLR* polymorphisms to the occurrence of infection [14, 25]. Our recent study in fetuses and neonates, with and without congenital HCMV infection, presented that *TLR4* and *TLR9* SNPs were associated with the development of congenital cytomegaly [14]. Considering the multiple-SNP analysis, performed in the current study for *TLR2*, *TLR4* and *TLR9* SNPs, a correlation was found between the presence of AA complex variants for *TLR2* 2258 and *TLR9* 2848 G>A SNPs and the occurrence of HCMV congenital infection. Moreover, among the infected offsprings, the AA multiple-SNP variants were significantly associated with higher viral loads, estimated in maternal blood and urine samples. The observed increased viral levels in fetal amniotic fluids and maternal urine samples were also correlated with the occurrence of GA complex variants for the analyzed *TLR2* and *TLR9* SNPs among congenitally infected fetuses and neonates. Regarding these two polymorphisms, a previous study, performed for Polish infants with postnatal or unproven congenital HCMV infection, showed both heterozygotes and recessive homozygotes in *TLR9* -1486 T>C and 2848 G>A SNPs to be at almost 4-fold increased risk of HCMV disease in an adjusted model, including HCMV DNA loads [51]. Considering our outcomes, the common contribution of *TLR2* 2258 and *TLR9* 2848 G>A polymorphisms to the development of congenital infection seems to be particularly possible, since *TLR9* 2848 G>A SNP was also previously reported to be involved in an increased risk of HCMV infection among fetuses and neonates, although the polymorphism is not associated either with amino acid changes of TLR9 molecule or with alterations of the regulatory site of *TLR9* gene [14, 52]. Hence, the presented data suggest some role of different TLR molecules, as well as of various genetic modifications, located within *TLR* genes, in the occurrence of congenital HCMV infection.

Since *TLR2* 2258 G>A SNP coding Arg753Gln non-synonymous change is located within a group of highly conserved amino acids at the C-terminal cytoplasmic Toll-interleukin 1 receptor domain of the TLR2 molecule, its contribution to the receptor-induced signal pathways was reported as plausible [53, 54]. Accordingly to molecular modeling studies of *TLR2* Arg753Gln variation, discrete main and side chain differences were reported, affecting the analyzed residue itself [54]. The alteration was suggested to be associated with interactions between the intracellular signaling of Toll-IL-1R (TIR) 2 and TIR1 domains [54]. In addition, the polymorphism was determined to change the electrostatic potential of the DD loop and αD region, related to the Arg753Gln polymorphism, causing a slight movement of the residues, participating in protein-protein interactions [54]. *TLR2* 2258 G>A SNP was reported as correlated with impaired agonist-induced tyrosine phosphorylation, heterodimerization of TLR2 with TLR6, and recruitment of Mal and MyD88 adapter proteins [54]. Out of them, Myd88 is the key molecule involved in the transmission of TLR2 induced signaling pathways of non-specific anti-HCMV response [54]. Hence, it is plausible that *TLR2* 2258 G>A polymorphism, may be involved in the occurrence and development of congenital infection with HCMV through affected TLR2/Myd88 signaling, caused by altered conformation and the electrostatic potential of the TIR2 domain [54]. In transfected HEK293 cells, treated with *Mycobacterium tuberculosis* or mycobacterial components, the presented signal alterations were associated with a decreased phosphorylation of p38, NF-κB activation and IL8 transcription [54]. Another in vitro study, performed with transfected HEK293 cells, challenged with a tripalmitoylated hexapeptide (Pam3CSK4), showed *TLR2* 2258 G>A SNP to have been associated with a substantially reduced activity of TLR2, as well as with 50% decreased activity of the NF-κB-driven reporter gene [55]. In other studies, the heterozygotic status in *TLR2* 2258 G>A locus was also reported to be associated with the molecule hypo-responsiveness upon stimulation with the synthetic TLR2 ligand Pam3CysSK4, as well as reduced signaling via the TLR2/TLR1-complex [38, 56]. Considering the previous data on the function of TLR2 and its 2258 G>A polymorphism, in the related signaling pathways, as well as the outcomes of our study, we suggest that, in Polish fetuses and newborns with congenital HCMV infection, GA heterozygotic status in the analyzed region may cause hypo-responsiveness of the produced TLR2 molecule to infection with HCMV. The altered function of TLR2 may result from impaired heterodimerization of TLR2 with TLR1 molecule, the affected TLR2/Myd88 signaling, the changed TLR2-dependent NF-κB signaling and, in result, the inflammatory cytokine release. It seems possible that in GA heterozygotes, the presence of only one correct G allele in *TLR2* 2258 G>A locus might be insufficient for complete immune response against HCMV. Considering all the recently obtained results for *TLR2*, *TLR4* and *TLR9* SNPs, the *TLR2* 2258 G>A polymorphism seems to be an important genetic factor, correlated with an increased risk of HCMV congenital infection among fetuses and neonates. However, further studies would be justified to investigate in more detail the molecular mechanism which underlies *TLR2* polymorphism involvement in the development of congenital HCMV infection.

## Conclusions

The results of our study demonstrate that *TLR2* 2258 G>A SNP may be an important genetic factor of the previously analyzed *TLR2*, *TLR4* and *TLR9* polymorphisms, which is involved in the development of congenital HCMV infection in Polish fetuses and neonates. The GA heterozygotic status in the polymorphic region was correlated with HCMV infection, increasing 10 times the risk of the infection and the A allele in *TLR2* SNP was significantly more frequently found among the infected fetuses and neonates than in the uninfected controls. Moreover, the occurrence of AA complex variant in the range of both *TLR2* 2258 and *TLR9* 2848 G>A SNPs, was correlated with an increased risk of the studied congenital infection as well. Taking into account the HCMV DNA loads, significantly higher levels were determined in the amniotic fluids of fetuses with GA multiple-SNP variants for the analyzed *TLR2* and *TLR9* polymorphisms, in maternal blood specimens of mothers for the offsprings of AA complex variants, as well as in maternal urine samples for the carriers of GA multiple-SNPs. TLR2 molecule was previously reported to have been associated with altered immune responses to HCMV, particularly in triggering the NF-κB activity and the release of inflammatory cytokines. An in vitro study with transfected HEK293 cells showed *TLR2* 2258 G>A SNP contribution to reduced activity of *TLR2* reporter gene. In case of congenital disease in Polish fetuses and neonates, the participation of the analyzed *TLR2* SNP in the development of HCMV infection seems to be fairly plausible through reduced TLR2 activity, impaired heterodimerization of TLR2 with TLR1 molecule, affected TLR2/Myd88 signaling as well as TLR2-dependent NF-κB molecule. Considering *TLR9* 2848 G>A SNP, the polymorphism has recently been determined to be correlated with an increased risk of congenital HCMV infection, while it is not associated with any molecular changes of the regulatory site within *TLR9* gene or the encoded protein. Therefore, the common contribution of *TLR9* 2848 G>A, together with *TLR2* 2258 G>A SNP, to the occurrence of HCMV infection seems possible. Further research would be beneficial with detailed mechanistic studies on the role of *TLR2* 2258 SNP in congenital infection with HCMV.
